# Meaningful messaging: Sentiment in elite social media communication with the public on the COVID-19 pandemic

**DOI:** 10.1126/sciadv.abg2898

**Published:** 2021-07-14

**Authors:** Janet M. Box-Steffensmeier, Laura Moses

**Affiliations:** Department of Political Science, The Ohio State University, 154 N. Oval Mall, Columbus, OH 43210, USA.

## Abstract

Elite messaging plays a crucial role in shaping public debate and spreading information. We examine elite political communication during an emergent international crisis to investigate the role of tone in messaging, information spread, and public reaction. By measuring tone in social media messages from members of the U.S. Congress related to the COVID-19 pandemic, we find clear partisan differences and a differential impact of tone on message engagement and information spread. This suggests that even in the midst of an international health crisis, partisanship and emotional rhetoric play a critical part in elite communications and contribute to the attention messages receive. The messaging on COVID-19 is polarized and fractured. The valenced messaging provokes divergence in public engagement, reaction, and information spread. These results have important implications for studies of representation, public opinion, and how government can effectively engage individuals in emergent situations or pivotal moments.

## Teaser

A new study finds how tone in elite messaging affects information spread and public reaction on social media during the pandemic.

## INTRODUCTION

Elite messaging has a critical role in framing policies and shaping public debates. Intuitively, we recognize that tone is a substantive and important aspect of messaging. The tone of a message can heighten anxieties or bolster public confidence in the information provided. Because the public depends on elite messaging for information, especially during crises, the use of a particular tone in messages can affect the response from the public and the spread of information. The public responds to not only the content but also the tone in messages sent by political elites.

The outbreak of coronavirus disease 2019 (COVID-19) is one of the gravest global public health and economic crises for the modern world. Messaging related to the pandemic is particularly important for public health, information sharing, and personal behavior. Historically, elites “rally around the flag” during times of crisis and suspend their adversarial behavior to share a unified message (*1*–*3*). However, heightened levels of polarization can fracture messaging and heighten the positivity or negativity officials relay in messaging. With a polarized and fractured response from elites, how does the public respond to the valence of information?

In the discipline of political science, how public engagement is influenced by tone and partisanship remains understudied. Few studies consider the relationship between communication effectiveness and sentiment in congressional communication. Tone is a vital component in framing and influencing the public within policy debates (*4*–*7*). Understanding how tone affects public reactions can provide an understanding of how elected officials use tone to frame their messaging. Studies of how members of Congress use the internet show its impact on congressional behavior (*8*, *9*). Members use social media and rely on these platforms for connecting with the public (*10*–*12*). Their presence on Facebook allows them to broaden their communication efforts and increase their connection to the public frequently and directly.

We investigate tone in messages, information spread, public reaction, and how these aspects of messaging may be related to communication effectiveness by using a comprehensive dataset of Facebook posts on COVID-19 from members of Congress. We measure the tone in members’ Facebook messages using a state-of-the-art machine learning model to predict sentiment scores. These measures allow us to examine the dominant tone in communications and how tone and political factors, including ideology and electoral competitiveness, contribute to public reaction and information spread online. We then couple these tone measures with topic model results to identify the predominant subjects of these messages and the associated tone along party lines. We also identify shifts in tone over time. Despite being an international crisis, the messaging on COVID-19 is polarized and fractured. We find that partisan tone differences are evident across message content, even at the outset of the crisis. The valenced messaging provokes divergence in public engagement, reaction, and information spread.

## RESULTS

**Fig. 1:**
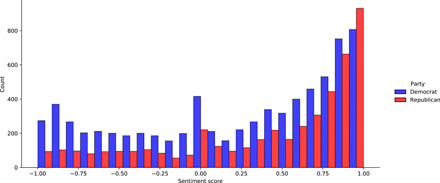
Distribution of sentiment scores across posts.

The descriptive statistics illustrate some key characteristics about tone in elite messages. Figure 1 shows the frequencies of sentiment scores across the Facebook posts by party. Sentiment scores range from −1 to 1, where −1 is the most negative and 1 is the most positive (see Materials and Methods for more details). There are clear partisan differences in the use of tone in messages about the pandemic. This partisan divergence is supported by related findings on polarization in messaging on other social media platforms (*8*, *13*).

Democrats posted about COVID-19 more than Republicans, averaging 26 posts per member, while Republicans averaged 18 posts per member during the period of study. The prevalence of messages on the pandemic fits with the professed electoral strategies of both parties (*14*, *15*). Figure 1 illustrates that, on average, Republicans used a more positive tone and Democrats used a negative tone. This is evident in the heavy rightward skew of the Republican tone distribution when contrasted with the tone distribution for Democrats. Both Republicans and Democrats skewed right, meaning that members of both parties used a more positive tone overall. Democrats were far more likely to use neutral language, as noted by the density of sentiment scores closer to zero. This suggests that Republicans invoked valenced messaging about the pandemic more so than Democrats.

### Social media engagement, spread, and emotional reaction

**Fig. 2:**
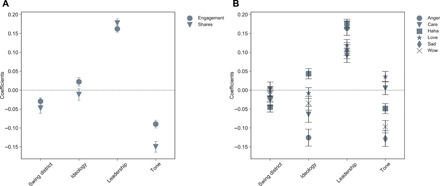
Multiple regression results for tone. (**A**) Coefficients for engagement and spread with 95% confidence intervals. (**B**) Coefficients for emotional reactions with 95% confidence intervals.

Investigation of public engagement, emotional reactions, and information spread reveals additional evidence of partisanship and clarifies the role of tone in messaging. Social media posts can be reacted to with *likes*. In addition, users may respond using a variety of emoticons such as *Wow*, *Anger*, and *Love* to express emotional reactions. Posts are organically spread through social networks by *sharing*. For each post, we predicted the tone in the message and evaluated the explanatory power of tone for engagement, information spread, and emotional reactions using multiple regression models. Figure 2 shows the results of the multiple regression models. We used robust standard errors because there are multiple posts from the same legislator in this dataset, which creates a hierarchical structure. This methodology is appropriate given the large number of politicians reflected in the data (*8*, *16*). Reestimation was carried out with regular standard errors, and the results indicated consistent results (see the Supplementary Materials for details and robustness checks). Separate features in the analysis account for district and member-related characteristics that relate to public engagement and reaction. We included member ideology from *Voteview*, where values closer to −1 are more liberal and values closer to 1 are more conservative. We also include binary variables to indicate whether a district was swing district, wherein the margin of victory was within 5%, and leadership status. The features are scaled and standardized. The dependent variables are overall engagement and the shares a given post receives, as shown in Fig. 2A, and the specific emotional reaction to the post, as illustrated in Fig. 2B.

Figure 2 shows that tone affects the engagement and spread of a member’s message. A more negative tone increases the reaction and spread of information. Leadership and ideology also contribute to engagement and spread. Negatively valenced messages received the most spread. Conservative members see an increase in information engagement, while more liberal members see a slight increase in information sharing. Figure 2B shows the results for predicting the number of emotional reactions. In these results, we see the expected positive tone predicting positive reactions like *love* and negative tone increasing *angry* or *sad* reactions. Negative tone also increases the *haha* reactions, suggesting that partisanship or reactions using this emotion imply sarcasm or distaste. There is an increase in *sad* reactions the more negative the tone and liberal the member.

### Polarization in tone and topic

Congressional communication engagement is further increased by ideology; the more conservative a member is, the more engagement there is with their content. Member ideology affects engagement with their messaging. Ideological positioning does not correlate with a more negative or positive tone in messaging. While conservatives have more overall engagement, liberal members are more likely to see their information shared. When the tone is more negative, there is an increase in the expected shares. Overall, COVID-19 message engagement and information spread are related to both partisanship and the tone that members use in their communications.

**Fig. 3:**
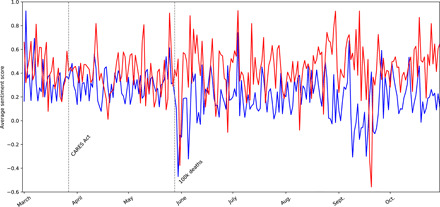
Average sentiment from March to October 2020.

Figure 3 reveals the daily average sentiment by political party. At the beginning of the pandemic, the average tone between the two parties was more similar. Over time, even with the fluctuations in averages, there is a consistent and clear difference where Democrats continue to use a more neutral and negative tone than Republicans throughout the period of study. The timeline illustrates some variation, and it is tempting to consider that tone in communications related to COVID-19 is deliberately tied to other domestic political events, e.g., we see that both parties use a more positive tone just before and after the passage of the CARES Act. Democrats’ tone shifts drastically relative to Republicans’ tone after events marking the severity of the pandemic, including the deaths of 100,000 people.

**Fig. 4:**
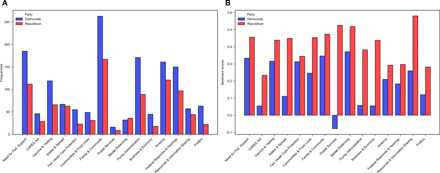
Tone and topics in COVID-19 messages. (**A**) Frequency of topics by party. (**B**) Average topic sentiment by party.

Next, we turned to analysis of tone and message content. Figure 4A shows the results of the topic model, displaying the frequency of topics, and Fig. 4B shows the average topic sentiment score by party. The partisan differences in messaging about the pandemic demonstrate how Democrats overwhelmingly discuss COVID-19 more and use a more negative tone when communicating about almost every COVID-19–related topic. The evidence suggests that polarization plays a role in the tone and how partisans talk about the pandemic.

Overwhelmingly, there is a partisan pattern to the tone and topics of elite messaging about the crisis, as seen in Fig. 4. Democrats are also more likely to discuss the federal response and use a negative tone in doing so. Of particular political note, the *Federal Response & Hearings* topic includes the work of congressional committees and Dr. Fauci, while *Trump Administration* focuses on President Donald Trump’s action, inaction, and general response to the pandemic (see the Supplementary Materials for additional information on topics). Republicans speak more favorably about the federal plan and response.

The tone and topics revealed by this analysis are telling about elite discussion around the pandemic. Where one might expect a more objective response to the scientific information, this analysis shows clear partisan differences in the rhetoric about the pandemic. Democrats discuss every topic more except social distancing, where there is a slight Republican edge. A similar disparity in the average tone of politicians’ messages overall is evident. Democrats use a decidedly more negative tone in their posts regarding the *Trump Administration*, which is also a subject discussed much more frequently by Democrats than Republicans.

The most positive topic on average for Republicans is *Resources and Information Sharing*. The most negative topic on average for Democrats is the *Postal Services* topic, which is related to the post office’s role in voting and timely deliveries of medicines. Donald Trump admitted to blocking funding for the postal service, and these discussion topics provide further evidence on how the topic, *Politics*, is related to the election and other domestic political events. These findings about content and tone demonstrate that members of Congress were perpetuating valenced tone, directly tying messages about COVID-19 to other partisan issues.

## DISCUSSION

Our analysis of elite messages demonstrates that the associated tone was polarized from the outset of public discussion about the pandemic. The valence and context of information related to the crisis suggest that political elites crafted messaging to represent their opinions and emphasize political identity, rather than sending a unified government response. In our analysis of COVID-19 communication, there is not a “rally around the flag effect” by U.S. political leaders, suggesting a shift toward partisan politics. When communicating about COVID-19, most of the posts by members of Congress are valenced, rather than taking on a unified or neutral tone. Using positive and negative tones may be signaling messages to the relevant in-group or out-group to enhance one’s identity. Elites may use tone strategically to foster support for pandemic actions, but they also leverage sentiment for political advantage. These results indicate that members’ communications are polarized even around an emergent, life-threatening disease. The divergence in tone and messages shared online by elites may be related to the fractured public reaction to the crisis. It also indicates the crucial role that tone has in elite cues, which anchor the public’s attitudes and reactions (*17*, *18*).

The tone elites use affect the public reaction and engagement with messaging. Negative tone leads to an increase in engagement for both parties, even more than some partisan factors. While members in contested districts have decreased attention and reaction given to their posts, the tone of members’ Facebook communications has a greater impact than some ideological factors in predicting engagement. Negative tone increases the spread of their messages online, which may be useful for creating effective content. This contributes to the understanding of information diffusion, showing that information with a negative tone spreads more on social media. The specific emotional reaction metrics provide insights to public response by providing a more nuanced understanding and revealing some of the self-presentation choices the public makes when engaging with messaging from elites. The evidence on messaging presented here suggests that on other issues, the tone that congressional communications use can affect the spread, reaction, and engagement by the public to the posts made online.

This analysis has not accounted for all the sources of tone, as it only accounts for the tone in the text and not any associated images, videos, or other media that may accompany a post. There may well be an additive or competing signal from different components of a social media message that generates important influence for the public. While we know that congressional offices value social media, the messaging choices likely vary, so our inferences about engagement strategy are conjectures. For example, some posts were lengthy excerpts from congressional newsletters or the text of statements made on the Senate floor, and others were short texts crafted specifically for sharing on social media. Despite these limitations, our findings show that Democrats and Republicans send divergent cues and tone across topics used in the discussion of the COVID-19 crisis.

These findings are the first of their kind to consider how sentiment plays a role in engaging constituents on social media. How tone affects engagement with elite communication has important implications for studies of representation, public opinion, and how government can effectively engage individuals with crucial information in emergent or pivotal moments. Tone provokes divergence in partisan cues and information content. It also conveys values and reveals the choices elites make in representing themselves online. Messaging on Facebook suggests that members of Congress are not amplifying the public health or medical professional information about the global health emergency. Understanding the effect of tone on information engagement and spread is critical for crafting effective messaging and sharing critical information. Public crises, like the pandemic, highlight how central social media is to messaging and information, and how important it is to understand the mechanisms that increase interaction and sharing of messages. These results further our understanding of how emotions correlate with spread and engagement in social media cues from elites, helping to shape our understanding of emotional importance in messaging.

## MATERIALS AND METHODS

We curated a list of all Facebook pages affiliated with members of the 116th Congress. This included official congressional member pages and verified personal or campaign pages that some members maintain in addition to their official pages. The inclusion of additional verified Facebook pages is necessary to fully capture communication because some member’s official district pages are less active than their verified personal pages. We collected their posts about COVID-19 via CrowdTangle, a public insights tool owned and operated by Facebook. Between 1 March and 30 October 2020, members generated 12,031 posts about COVID-19. To identify posts about the pandemic, we created a dictionary of keywords related to COVID-19 and then applied a set of preprocessing steps that included tokenizing the text and omitting rarely used or frequently used tokens (details are in the Supplementary Materials). We then combined the Facebook information with data on the members, such as partisanship and ideology.

Then, we conducted sentiment analysis on the messages to evaluate tone. Specifically, we used VADER (Valence Aware Dictionary and sEntiment Reasoner) to evaluate the sentiment of the message (*19*). VADER was developed specifically for measuring tone along positive, negative, and neutral dimensions in social media corpora. VADER is unique in using the sentiment of particular words as well as accounting for word-order relationships and particularities of abbreviations. This is an extensively validated tool for social media analysis and generates some of the most accurate classifications in a benchmark of sentiment analysis tools (*20*–*22*). Our validation of the VADER sentiment scores in our dataset was done by sampling posts from the bottom, median, and top 10% of VADER scores and having student coders hand-label the tone. We achieve an average Cohen’s κ of 0.79 among the hand-labeled messages. The hand-labeled scores predicted VADER scores with an accuracy rate of 0.76 (details are in the Supplementary Materials). We then used these VADER sentiment scores to evaluate the relationship between message tone, partisanship, and engagement. We measure engagements and information spread using the available metadata from posts. Engagement is measured as the sum of interactions with a post (likes, emotional reactions, or comments). Information spread is measured as the total number of shares. To minimize the role of outliers due to the popularity of a particular member’s page, the number of engagements and the number of shares with each post are transformed to a logarithmic scale.

Next, we fit a topic model to analyze the messages and to determine clusters of words. Specifically, we fit a Latent Dirichlet Allocation (LDA) topic model (*23*) using a split-sample approach. We fitted and tuned the model to a training set and then used this model to predict the topics of the remaining posts. The fit of the LDA model is evaluated using coherence, which measures the degree of semantic similarity between high-scoring words of topics. This provides an indicator of the best topic number for model interpretation while minimizing statistical artifacts (*24*).

## SUPPLEMENTARY MATERIALS

Supplementary material for this article is available at http://advances.sciencemag.org/cgi/content/full/7/29/eabg2898/DC1
